# MiR-29c-3p May Promote the Progression of Alzheimer's Disease through BACE1

**DOI:** 10.1155/2021/2031407

**Published:** 2021-12-15

**Authors:** Yanqun Cao, Xiangxiang Tan, Quzhe Lu, Kai Huang, Xiaoer Tang, Zhiming He

**Affiliations:** ^1^Shaoyang University Basic Medical College, Shaoyang 422000, Hunan, China; ^2^Shaoyang University School of Nursing, Shaoyang 422000, Hunan, China

## Abstract

The aim of this study was to explore the specific role of miR-29c-3p in Alzheimer's disease (AD). Animal models of AD were established by injecting streptozotocin (STZ) into mice through the lateral ventricle, while cell models of AD were induced by 10 *μ*M *β*-amyloid (A*β*). We detected miR-29c-3p and *β*-site amyloid precursor protein cleaving enzyme 1 (BACE1) contents and measured AD cell proliferation and apoptosis. A low miR-29c-3p level and a high BACE1 level were detected in the brain tissue of AD animal models and AD cell models. A*β*-processed cells had markedly lower proliferation activity, higher apoptosis, increased phosphorylation of tau protein was over phosphorylated, but the overexpression of miR-29c-3p or the silencing of BACE1 significantly enhanced the cell proliferation activity and reduced cell apoptosis by regulating the contents of related proteins. Inhibition of miR-29c-3p or overexpression of BACE1 aggravated A*β*-induced side effects. We used Targetscan7.2 to predict the downstream target genes of miR-29c-3p. Then, we detected that there were target binding sites between miR-29c-3p and BACE1. The rescue experiment identified BACE1 as a functional target for miR-29c-3p. AD leads to decreased miR-29c-3p level and increased BACE1 level. MiR-29c-3p has specific binding sites with the 3′-untranslated region (3′-UTR) of BACE1 and thus negatively regulates the BACE1 level, thereby affecting the progression of AD.

## 1. Introduction

Alzheimer's disease (AD), a neurodegenerative disease common in old people, mainly manifests as impaired cognitive functions, social disorders, abnormal behaviors, and memory loss [1, 2]. People with AD account for more than 80% of cases of dementia in people over 65 worldwide [3]. Currently, the number of AD patients in the United States exceeds 5 million, with an expected increase to 16 million by 2050 [4]. Decades of ongoing investigations of AD have not figured out its etiology. So far, there is no optimal treatment strategy for AD. The occurrence of AD is related to *β*-amyloid (A*β*) plaques and neurofibrillary tangles (containing tau protein) in the cerebral cortex and subcortical regions, so there is the speculation that the reducing or eliminating of this substance from the brain may prevent or reverse the progression of AD disease [4, 5].

MicroRNAs (miRNA), a class of endogenous noncoding RNAs with 18–24 nucleotides, play vital roles in the mammalian brain [6]. MiRNAs are highly expressed or specifically expressed in the nervous system to affect neural development and synaptic functions and participate in processes such as memory formation and regulation of protein synthesis, as well as to induce specific neurodegenerative diseases [7–9]. MiR-29c-3p is abnormally expressed in a variety of diseases [10]. With markedly lower expression in hepatocellular carcinoma, miR-29c-3p can inhibit tumors by targeting DNA methyltransferase 3B (DNMT3B) and the Hippo signaling pathway involving large tumor suppressor kinase 1 (LATS1) [11]. So far, little has been known about the effect of miR-29c-3p on AD progression. One of the main pathological changes caused by AD is senile plaque deposition in the brain, which mainly consists of A*β* that comes from amyloid precursor protein (APP) after being sequentially cleaved by *γ*-secretase and *β*-site amyloid precursor protein cleaving enzyme 1 (BACE1), also called *β*-secretase 1, a key gene for A*β* accumulation in the brain of AD patients [12, 13]. MiR-29C is involved in AD progression and reduces the accumulation of A*β* by decreasing the expression of BACE1 [14]. We need to study further to check if miR-29c-3p has the same function as miR-29c in AD patients.

Here, we assessed the function of miR-29c-3p in the proliferation and apoptosis of A*β*-induced AD cells, aiming to figure out the specific mechanisms of miR-29c-3p in AD.

## 2. Methods

### 2.1. Animal Models

We purchased male SPF C57BL/6J mice (20–25 g) from the Department of Laboratory Animal Science of China Medical University. After adaptive feeding for 1 week, we randomly assigned mice to the AD group (10 mice) and the control group (10 mice). Mice in the control group were reared normally, while mice in the AD group were made into AD models by injecting streptozotocin (STZ) through the lateral ventricle [15]. This study was carried out after obtaining approval from the ethics committee of our hospital, in strict accordance with guidelines issued by the Care and Use of Laboratory Animal and the National Institutes of Health (NIH).

### 2.2. Cell Culture and Transfection

We purchased PC12 cells from Shanghai Aulu Biological Technology Co., Ltd. (article number XB-2198) and cultured them in a DMEM medium (KL-P0032, Merck/sigma, Germany) with 10% fetal bovine serum at 37°C, with 5% CO_2_. We induced AD cell models with 10 *μ*M A*β* [16]. Cells were divided into control group, A*β* group, A*β* + miR-29c-3p inhibitor group, A*β* + miR-NC group, A*β* + miR-29c-3p mimics group, A*β* + sh-BACE1 group, A*β* + vector group, and A*β* + si-BACE1 group. We used the Lipofectamine™ 2000 kit to transfect plasmids into cells.

### 2.3. Quantitative Real-Time Polymerase Chain Reaction (qRT-PCR) Detection

We extracted the total RNA with the Trizol reagent (Thermo Fisher Scientific, Inc. Waltham, MA, USA) and measured its purity and concentration with a DR5000 UV-Vis spectrophotometer (BioRad, Hercules, CA, USA). We used the TaqMan miRNA reverse transcription kit (Thermo Fisher Scientific, Inc. Waltham, MA, USA) to perform the reverse transcription of RNA into cDNA. Reverse transcription and PCR amplification and quantification of RNA were performed on SYBR Premix Ex Taq™ (Takara, Shiga, Japan) and the PCR instrument produced by Applied Biosystems (item number: 7500). The design and synthesis of primers were completed by Sangon Biotech (Shanghai) Co., Ltd. BACE1: sense primer, 5′-CACTCTGTTCTGGGTGGTCC-3′; antisense primer, 5′-CATGGGGGATGCTTACCAGG-3′. Internal reference (GAPDH): sense primer, 5′-CGGATTTGGTCGTATTGGG-3′; antisense primer, 5′-CTGGAAGATGGTGATGGGATT-3′. MiR-29c-3p: sense primer, 5′-GAAGCACCATTTGAAATCAG-3′ and antisense primer, 5′-TTGGCACTAGCACATT-3′. Internal reference (U6): sense primer, 5′-CTTCACGAATTTGCGTGTCAT and antisense primer, 5′-GCTTCGGCAGCACATATAC-3′. PCR conditions: predenaturation at 95°C for 10 min, then denaturation at 95°C for 15 s, and annealing and extension at 60°C for 60 s. The results were calculated using 2^−∆∆ct^.

### 2.4. Western Blot Detection

The protein in cells was lysed by RIPA buffer (Cell Signal Technology, Inc., Danvers, MA, USA). We quantified the protein concentration with the BCA kit (Beyotime Biotechnology, Shanghai, China). The electrophoretic separation of proteins was performed using the 12% SDS-PAGE gel. Then, we transferred the protein to a PVDF membrane manufactured by Millipore and blocked the membrane in skimmed milk-PBS solution at room temperature for 1 hour. After the incubation of the membrane and the primary antibody (Abcam, Branford, CT, USA) at 4°C for one night, the primary antibody was washed off and then the horseradish peroxidase-labeled goat anti-rabbit secondary antibody (Abcam, Branford, CT, USA) was added to the membrane and incubated at 37°C for 1 hour. Finally, the membrane was subjected to a PBS washing, followed by color development using ECL luminescent reagent (Thermo Fisher Scientific, Waltham, MA, USA). The relative protein expression = gray value of the strip to be tested/gray value of the internal reference protein.

### 2.5. Detection of Cell Proliferation Activity

We employed an MTT kit (Thermo Fisher Scientific Co., Ltd., Hangzhou, China) to test the PC12 cell proliferation activity. Cells were incubated at 37°C with 5% CO_2_ for 24 h, 48 h, 72 h, and 96 h. Then, the medium was discarded and 20 *μ*L of MTT was added to per well for incubation for 4 hours. Next, we added 150 *μ*l of dimethyl sulfoxide to the well and shook the plate for 5–10 minutes to make sure the purple crystals were completely dissolved. Multiskan™ GO full-wavelength microplate reader (Thermo Fisher Scientific Co., Ltd., Hangzhou, China) was employed to test the absorbance at 450 nm to detect cell proliferation. The experiment was repeated 3 times.

### 2.6. Detection of Cell Apoptosis

We purchased an Annexin-V-FITC/PI Apoptosis Detection Kit (Thermo Fisher Scientific, Waltham, MA, USA) to test cell apoptosis. Then, we washed cells transfected with different plasmids with the cold phosphate saline buffer and trypsinized cells and collected them into a centrifuge tube. Twenty *μ*l of Annexin-V-FITC labeling solution, 1 ml of buffer, and 20 *μ*l of PI reagent were mixed together for incubation at room temperature for 5 minutes away from light, and then, cell apoptosis was detected on the FACScan flow cytometer (Becton Dickinson, USA).

### 2.7. Dual-Luciferase Reporter (DLR) Assay

The prediction of the binding sites between miR-29c-3p and BACE1 was performed on TargetScan. FragmentS of either the wild type (BACE1-wt) or mutant (BACE1-mut) BACE1 3′-untranslated region (3′-UTR) containing the predicted binding sites were cloned on the vector. After the DNA sequencing for verification, we transfected miR-29c-3p mimics or miR-NC into PC12 cells using a Lipofectamine™ 2000 kit (Invitrogen, USA). We harvested cells 48 hours later and conducted the DLR assay on DLR® (Promega Corporation).

### 2.8. Statistical Analysis

The statistical analysis was performed on the SPSS20.0 and the data visualization on the GraphPad 7. The intergroup comparison was analyzed by the independent *t*-test and the comparison between multiple groups by the one-way analysis of variance, with the LSD *t*-test as the post hoc test. *P* < 0.05 indicates a statistical difference.

## 3. Results

### 3.1. Effect of AD on MiR-29c-3p and BACE1 Levels

**Figure 1:**
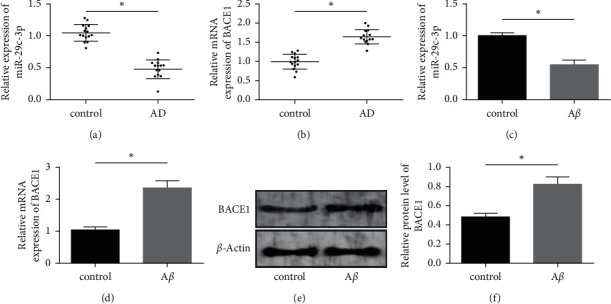
Effect of AD on miR-29c-3p and BACE1 levels. (a) MiR-29c-3p level in AD mouse models. (b) BACE1 level in AD mouse models. (c) MiR-29c-3p level in A*β*-processed PC12 cells. (d) BACE1 mRNA level in A*β*-processed PC12 cells. (e) BACE1 protein content profile in A*β*-processed PC12 cells. (f) BACE1 protein expression in A*β*-processed PC12 cells. Note: “^*∗*^” indicates  ^*∗*^*P* < 0.05 for the comparison between two groups.

The qRT-PCR detection revealed a low miR-29c-3p level and a high BACE1 level in brain tissues of mouse models (*P* < 0.05), a markedly reduced miR-29c-3p level, and markedly increased levels of BACE1 mRNA and protein in A*β*-processed PC12 cells (*P* < 0.05). More details are shown in Figure 1.

### 3.2. Function of MiR-29c-3p in the Apoptosis and Viability of A*β*-Induced PC12 Cells

**Figure 2:**
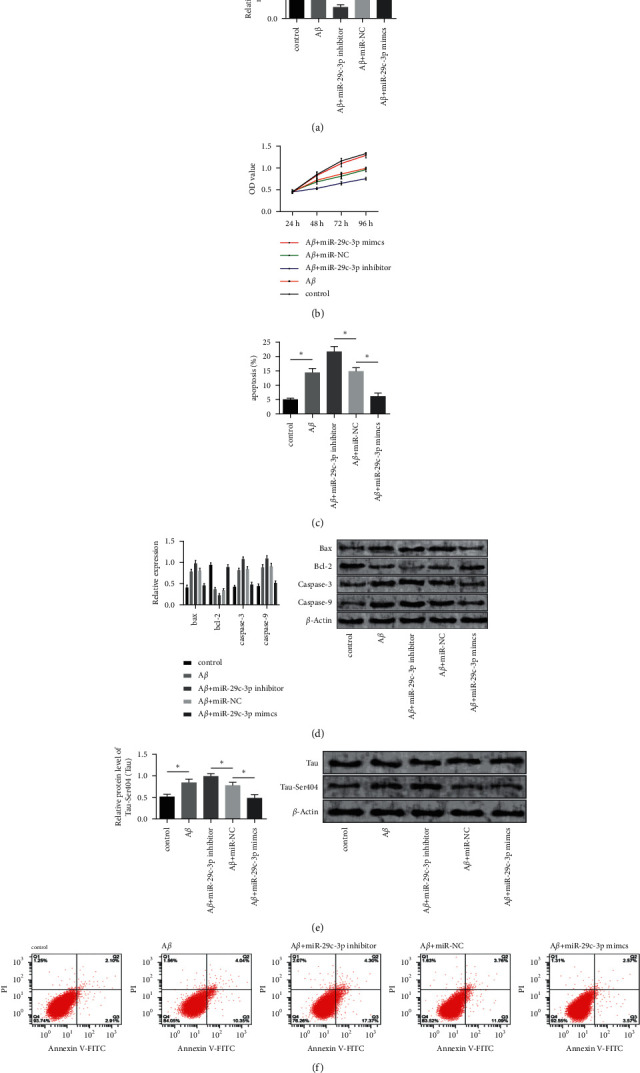
Function of miR-29c-3p in the apoptosis and viability of A*β*-induced PC12 cells. (a) miR-29c-3p level in cells after the transfection. (b) Cell proliferation activity after the transfection. (c) Cell apoptosis rate after the transfection. (d) Expression and content of apoptosis-related proteins after the transfection. (e) Tau protein phosphorylation after the transfection. (f) Cell apoptosis after the transfection. Note: “^*∗*^” indicates  ^*∗*^*P* < 0.05 for the comparison between two groups.

We transfected PC12 cells with miR-29c-3p inhibitor, miR-NC, and miR-29c-3p mimics separately. More details are shown in Figure 2. A*β*-processed cells had markedly lower cell viability and increased tau protein phosphorylation than cells from the control group (*P* < 0.05). MiR-29c-3p mimics relieved the toxic side effects caused by A*β*, enhanced cell viability, and reduced the level of tau protein phosphorylation, while miR-29c-3p inhibitor further inhibited cell viability (*P* < 0.05). A*β*-processed cells had markedly higher cell apoptosis, higher expression levels of apoptosis-related proteins (caspase-9, caspase-3, and Bax), and lower expression and content of Bcl-2 than cells from the control group (*P* < 0.05). The overexpression of miR-29c-3p attenuated the effects of A*β*, while the inhibition of miR-29c-3p accelerated the A*β*-induced cell apoptosis.

### 3.3. Effect of BACE1 on the Apoptosis and Viability of A*β*-Induced PC12 Cells

**Figure 3:**
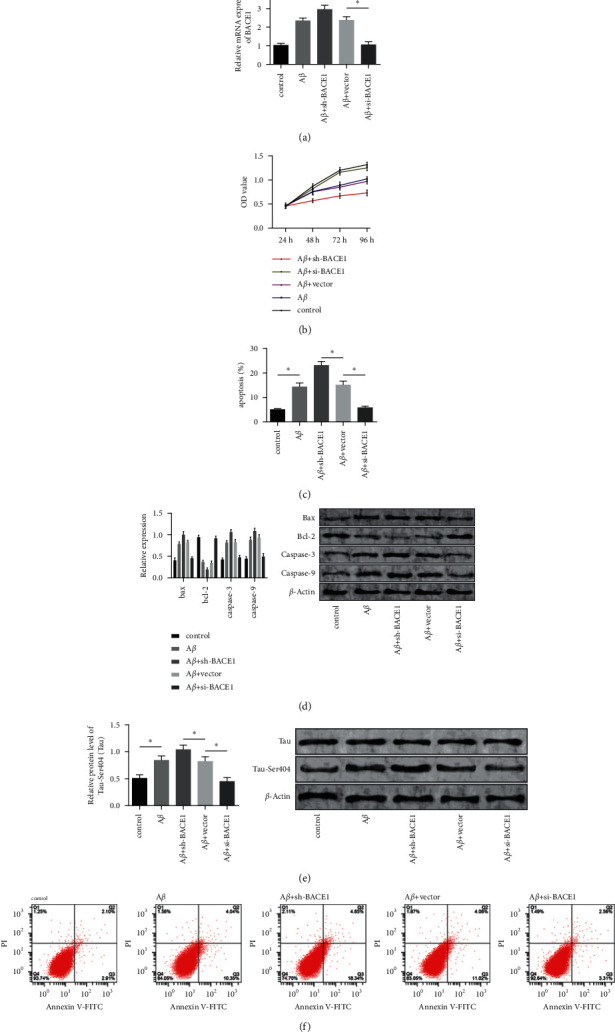
Function of BACE1 in the apoptosis and viability of A*β*-induced PC12 cells. (a) BACE1 level in cells after the transfection. (b) Cell proliferation activity after the transfection. (c) Cell apoptosis rate after the transfection. (d) Expression and content of apoptosis-related proteins after the transfection. (e) Tau protein phosphorylation after the transfection. (f) Cell apoptosis after the transfection. Note: “^*∗*^” indicates  ^*∗*^*P* < 0.05 for the comparison between two groups.

We transfected sh-BACE1, vector, and si-BACE1 into PC12 cells separately, as shown in Figure 3. Si-BACE1 relieved the toxic side effects caused by A*β*, enhanced cell viability, while sh-BACE1 further inhibited cell viability and reduced tau protein phosphorylation (*P* < 0.05). Cells in the si-BACE1 group had markedly lower cell apoptosis rate, lower content of caspase-9, caspase-3, and Bax, and higher content of Bcl-2 than cells from the control group (*P* < 0.05). Cells in the sh-BACE1 group had stronger apoptosis than cells in the A*β* group.

### 3.4. The Targeting Relationship between MiR-29c-3p and BACE1

**Figure 4:**
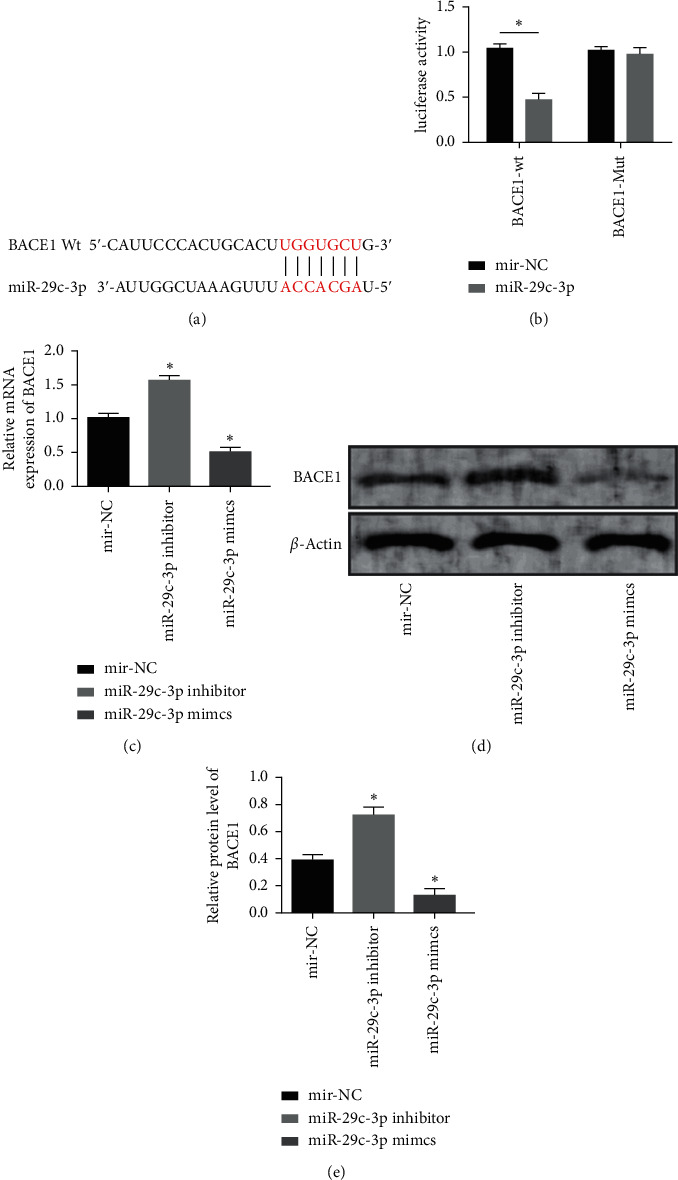
Targeting relationship between miR-29c-3p and BACE1. (a) Targeting binding sites between miR-29c-3p and BACE1. (b) Results of DLR assay. (c) BACE1 expression after the transfection. (d) BACE1 protein content after the transfection. (e) BACE1 protein expression after the transfection. Note: “^*∗*^” indicates  ^*∗*^*P* < 0.05 for the comparison with cells containing miR-NC.

We made a prediction of the target genes of miR-29c-3p on Targetscan7.2 and discovered targeted binding sites between the two genes. DLE assay revealed that the fluorescence activity of BACE1-Wt was significantly reduced. Transfection of miR-29c-3p inhibitor resulted in higher BACE1 protein level, while transfection of miR-29c-3p mimics resulted in lower BACE1 protein level (*P* < 0.05) (Figure 4).

### 3.5. Rescue Experiment

**Figure 5:**
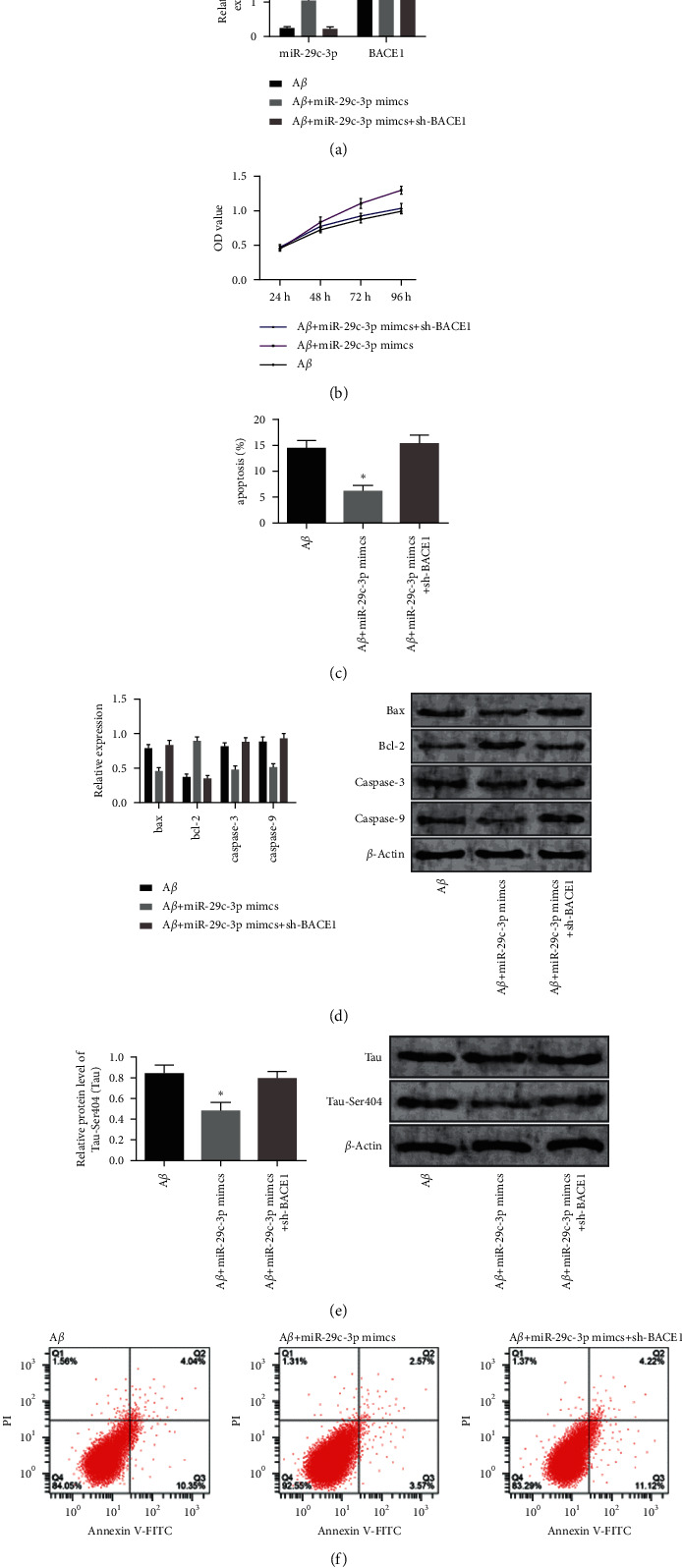
Rescue experiment. (a) MiR-29c-3p and BACE1 levels after the co-transfection. (b) Cell proliferation after the co-transfection. (c) Cell apoptosis rate after the co-transfection. (d) Expression levels and contents of apoptosis-related proteins after the co-transfection. (e) Tau protein phosphorylation after the co-transfection. (f) Cell apoptosis images by flow cytometry after the co-transfection. Note: “^*∗*^” indicates  ^*∗*^*P* < 0.05 for the comparison with cells containing miR-NC.

We transfected miR-29c-3p mimics and sh-BACE1 together into A*β*-induced PC12 cells and found no obvious difference in cell proliferation activity, cell apoptosis, and protein expression as compared with cells in the A*β* group (*P* > 0.05). Cells containing miR-29c-3p mimics and sh-BACE1 had a lower miR-29c-3p level, higher BACE1 level (*P* < 0.05), decreased cell viability, higher apoptotic rate, increased tau protein phosphorylation, higher expression levels of caspase-9, caspase-3, and Bax, and lower expression level of Bcl-2 compared with cells containing miR-29c-3p mimics (*P* < 0.05) (Figure 5).

## 4. Discussion

The pathogenesis of AD is caused by a variety of complex factors, such as age, heredity, and neurotransmission barriers [17, 18], bringing severe economic and living burdens to patients and society. The main pathological characteristics of AD disease include vascular amyloidosis, intracellular abnormal aggregation of phosphorylated tau protein, the formation of neurofibrillary tangles (NFTs), senile plaques formed by *β*-amyloid (A*β*) deposition, and neuron cells loss in the hippocampal area and cerebral cortex [19–21]. The accumulation and sedimentation of tau protein is an important factor responsible for neuronal degeneration and death, as well as the onset of AD [22]. The abnormal hyperphosphorylation of tau protein is caused by increased protein kinase activity or decreased phosphatase activity [23]. Therefore, reducing the abnormal hyperphosphorylation of tau protein is the key to treating AD.

In this study, the viability of PC12 cells processed by A*β* was markedly reduced, while the cell apoptosis was markedly increased, which is consistent with the results of previous researches [24, 25]. MiRNAs are closely related to the onset of AD and can affect cell differentiation, development, proliferation, and apoptosis by regulating the corresponding target genes [26, 27]. The abnormal expression of miRNAs in tissue or serum plays can affect the development and progression of diseases [28]. MiRNAs may affect the development and functions of the central nervous system [29]. MiR-29c-3p, a tumor suppressor [30], is remarkably downregulated in many solid tumors, including breast cancer [31], gallbladder cancer [32], and gastric cancer [33]. Sørensen et al. [34] proposed that the miR-29c-3p level is reduced in the cerebrospinal fluid of AD patients. Here, we detected a low expression level of miR-29c-3p in the brain tissue of AD mice and PC12 cell models of AD, which indicates that miR-29c-3p may affect the pathogenesis of AD. We transfected A*β*-processed PC12 cells with miR-29c-3p inhibitor, miR-NC, and miR-29c-3p mimics, separately. Then, we discovered that the inhibition of miR-29c-3p attenuated the reduction in cell viability and the rise in cell apoptosis induced by A*β*, while the overexpression of miR-29c-3p markedly offset the effects caused by A*β*, decreased the content of caspase-9, caspase-3, Bax, and enhanced the content of Bcl-2 (*P* < 0.05), further decreased cell viability, and enhanced the cell proliferation activity. Besides, miR-29c-3p overexpression relieved the abnormal phosphorylation of tau protein. MiR-10A can regulate neural cell proliferation and synaptic remodeling, as well as inhibit the BDNF-TrkB signaling pathway to promote cell apoptosis in AD mice [35]. MiR-200a-3p can reduce A*β*-induced PC12 cell apoptosis by targeting SIRT1 [36]. Based on the results of this study and previous findings, we speculate that miR-29c-3p can reduce the cytotoxicity caused by A*β* and induce AD lesions.

BACE1, a key enzyme to perform APP degradation to produce A*β*, has a markedly lower protein expression level in AD mice than in wild mice of the same age [16]. BACE1 protein levels and enzyme activity were remarkably higher in the brain samples of AD patients than in the brain samples of non-AD patients [37]. Many existing clinical drugs for AD aim to target decrease the protein level and enzyme activity of BACE1 to reduce A*β* production and secondary neuropathological changes [38]. Here, we detected a high BACE1 level in AD and found that BACE1 silencing relieved cytotoxicity caused by A*β*, decreased cell apoptosis, increased cell viability, and reduced the phosphorylation of tau protein. Many studies have shown that miRNAs play a part in the occurrence and development of AD through regulating BACE1. Feng et al. [39] proposed that miR-124 can reduce A*β*-induced inhibition of cell viability and decrease the apoptosis of SH-SY5Y cells by regulating BACE1. Bioinformatics analysis revealed that BACE1, a gene closely related to A*β* production in the course of AD, is a potential functional target gene for miR-29c-3p. In this study, the DLR assay verified the target gene of miR-29c-3p. BACE1-Wt fluorescence activity was significantly reduced, but BACE1-Mut had no significant change. The results of plasmid transfection revealed that miR-29c-3p overexpression inhibited the activity of BACE1, indicating miR-29c-3p has a negative regulatory effect on BACE1. The results of rescue experiments demonstrated that sh-BACE1 significantly reversed the therapeutic effect of miR-29c-3p mimics, identifying BACE1 as a functional target for miR-29c-3p.

## 5. Conclusion

This study mainly explored the underlying mechanism of miR-29c-3p in AD, aiming to provide new treatment targets for AD. However, this study is subject to some limitations. Given the complicated regulatory mechanism of AD, we did not figure out the specific regulatory pathways of AD. Besides, we will explore and the role of miR-29c-3p in the life and behavior of AD mice in the future to perfect this study.

In summary, AD can decrease the expression of miR-29c-3p and increase the expression of BACE1. MiR-29c-3p has specific binding sites with the 3′-untranslated region (3′-UTR) of BACE1 and thus negatively regulates the BACE1 level, thereby affecting the progression of AD.

## Data Availability

The datasets used and/or analyzed during the current study are available from the corresponding author upon reasonable request.

## References

[1] Hampel H., Mesulam M.-M., Cuello A. C. (2018). The cholinergic system in the pathophysiology and treatment of Alzheimer’s disease. *Brain*.

[2] Kelly S. C., He B., Perez S. E., Ginsberg S. D., Mufson E. J., Counts S. E. (2017). Locus coeruleus cellular and molecular pathology during the progression of Alzheimer’s disease. *Acta Neuropathologica Communications*.

[3] Reitz C., Mayeux R. (2014). Alzheimer disease: epidemiology, diagnostic criteria, risk factors and biomarkers. *Biochemical Pharmacology*.

[4] Blass B. E. (2019). Bridged piperidine derivatives useful as *γ*-secretase inhibitors for the treatment of Alzheimer’s disease. *ACS Medicinal Chemistry Letters*.

[5] Quiroz Y. T., Sperling R. A., Norton D. J. (2018). Association between amyloid and tau accumulation in young adults with autosomal dominant Alzheimer disease. *JAMA Neurology*.

[6] Nelson P. T., Wang W.-X. (2010). MiR-107 is reduced in Alzheimer’s disease brain neocortex: validation study. *Journal of Alzheimer’s Disease*.

[7] Schratt G. M., Tuebing F., Nigh E. A. (2006). A brain-specific microRNA regulates dendritic spine development. *Nature*.

[8] Luo L. (2002). Actin cytoskeleton regulation in neuronal morphogenesis and structural plasticity. *Annual Review of Cell and Developmental Biology*.

[9] Luo J.-Z., Zhu J., Zhang L. (2019). MiR-29a is down-regulated and inhibits cell proliferation in osteosarcoma:a meta analysis. *International Journal of Clinical and Experimental Medicine*.

[10] Fang R., Huang Y., Xie J., Zhang J., Ji X. (2019). Downregulation of miR-29c-3p is associated with a poor prognosis in patients with laryngeal squamous cell carcinoma. *Diagnostic Pathology*.

[11] Wu H., Zhang W., Wu Z. (2019). miR-29c-3p regulates DNMT3B and LATS1 methylation to inhibit tumor progression in hepatocellular carcinoma. *Cell Death and Disease*.

[12] Zhu H., Huang F., Li Z., Chen F., Xu Y. (2019). Genistein protects against rat hippocampus amyloid-*β*1-42 neurotoxicity through p-mTOR-dependent autophagy. *International Journal of Clinical and Experimental Medicine*.

[13] Al-Atrache Z., Lopez D. B., Hingley S. T., Appelt D. M. (2019). Astrocytes infected with Chlamydia pneumoniae demonstrate altered expression and activity of secretases involved in the generation of *β*-amyloid found in Alzheimer disease. *BMC Neuroscience*.

[14] Lei X., Lei L., Zhang Z., Zhang Z., Cheng Y. (2015). Downregulated miR-29c correlates with increased BACE1 expression in sporadic Alzheimer’s disease. *International Journal of Clinical and Experimental Pathology*.

[15] Nakhate K. T., Bharne A. P., Verma V. S., Aru D. N., Kokare D. M. (2018). Plumbagin ameliorates memory dysfunction in streptozotocin induced Alzheimer’s disease via activation of Nrf2/ARE pathway and inhibition of *β*-secretase. *Biomedicine and Pharmacotherapy*.

[16] Zhao M.-Y., Wang G.-Q., Wang N.-N., Yu Q.-Y., Liu R.-L., Shi W.-Q. (2019). The long-non-coding RNA NEAT1 is a novel target for Alzheimer’s disease progression via miR-124/BACE1 axis. *Neurological Research*.

[17] Teter B., Morihara T., Lim G. P. (2019). Curcumin restores innate immune Alzheimer’s disease risk gene expression to ameliorate Alzheimer pathogenesis. *Neurobiology of Disease*.

[18] Meyer P.-F., Savard M., Poirier J. (2018). Bi-directional association of cerebrospinal fluid immune markers with stage of Alzheimer’s disease pathogenesis. *Journal of Alzheimer’s Disease*.

[19] Villeneuve S., Vogel J. W., Gonneaud J. (2018). Proximity to parental symptom onset and amyloid-*β* burden in sporadic alzheimer disease. *JAMA Neurology*.

[20] Rzaewski K., Wang L., Haus J. W. (1989). Two-frequency above-threshold ionization with smooth pulses. *Physical Review A*.

[21] de Heus R. A. A., Olde Rikkert M. G. M., Tully P. J., Lawlor B. A., Claassen J. A. H. R., Group N. S. (2019). Blood pressure variability and progression of clinical Alzheimer disease. *Hypertension*.

[22] Harrison T. M., La Joie R., Maass A. (2019). Longitudinal tau accumulation and atrophy in aging and Alzheimer disease. *Annals of Neurology*.

[23] Ma R.-h., Zhang Y., Hong X.-y., Zhang J.-f., Wang J.-Z., Liu G.-p. (2017). Role of microtubule-associated protein tau phosphorylation in Alzheimer’s disease. *Journal of Huazhong University of Science and Technology-Medical sciences*.

[24] Zhao J., Lu S., Yu H., Duan S., Zhao J. (2018). Baicalin and ginsenoside Rb1 promote the proliferation and differentiation of neural stem cells in Alzheimer’s disease model rats. *Brain Research*.

[25] Ziegler-Waldkirch S., d’Errico P., Sauer J. F. (2018). Seed-induced abeta deposition is modulated by microglia under environmental enrichment in a mouse model of Alzheimer’s disease. *The EMBO Journal*.

[26] Lin Y., Liang X., Yao Y., Xiao H., Shi Y., Yang J. (2019). Osthole attenuates APP-induced Alzheimer’s disease through up-regulating miRNA-101a-3p. *Life Sciences*.

[27] Pogue A. I., Lukiw W. J. (2018). Up-regulated pro-inflammatory MicroRNAs (miRNAs) in Alzheimer’s disease (AD) and age-related macular degeneration (AMD). *Cellular and Molecular Neurobiology*.

[28] Swarbrick S., Wragg N., Ghosh S., Stolzing A. (2019). Systematic review of miRNA as biomarkers in Alzheimer’s disease. *Molecular Neurobiology*.

[29] Shu P., Wu C., Liu W. (2019). The spatiotemporal expression pattern of microRNAs in the developing mouse nervous system. *Journal of Biological Chemistry*.

[30] Schmitt M. J., Margue C., Behrmann I., Kreis S. (2013). MiRNA-29: a microRNA family with tumor-suppressing and immune-modulating properties. *Current Molecular Medicine*.

[31] Bhardwaj A., Singh H., Rajapakshe K. (2017). Regulation of miRNA-29c and its downstream pathways in preneoplastic progression of triple-negative breast cancer. *Oncotarget*.

[32] Shu Y.-J., Bao R.-F., Jiang L. (2017). MicroRNA-29c-5p suppresses gallbladder carcinoma progression by directly targeting CPEB4 and inhibiting the MAPK pathway. *Cell Death and Differentiation*.

[33] Matsuo M., Nakada C., Tsukamoto Y. (2013). MiR-29c is downregulated in gastric carcinomas and regulates cell proliferation by targeting RCC2. *Molecular Cancer*.

[34] Sørensen S. S., Nygaard A. B., Christensen T. (2016). miRNA expression profiles in cerebrospinal fluid and blood of patients with Alzheimer’s disease and other types of dementia - an exploratory study. *Translational Neurodegeneration*.

[35] Wu B. W., Wu M. S., Guo J. D. (2018). Retracted : effects of microRNA‐10a on synapse remodeling in hippocampal neurons and neuronal cell proliferation and apoptosis through the BDNF‐TrkB signaling pathway in a rat model of Alzheimer’s disease. *Journal of Cellular Physiology*.

[36] Zhang Q.-S., Liu W., Lu G.-X. (2017). miR-200a-3p promotes *β*-Amyloid-induced neuronal apoptosis through down-regulation of SIRT1 in Alzheimer’s disease. *Journal of Biosciences*.

[37] Pera M., Alcolea D., Sánchez-Valle R. (2013). Distinct patterns of APP processing in the CNS in autosomal-dominant and sporadic Alzheimer disease. *Acta Neuropathologica*.

[38] Tomita T. (2016). BACE1 inhibitors for the treatment of Alzheimer disease. *Nihon Rinsho*.

[39] An F., Gong G., Wang Y., Bian M., Yu L., Wei C. (2017). MiR-124 acts as a target for Alzheimer’s disease by regulating BACE1. *Oncotarget*.

